# Simple method for quantification of anionic biosurfactants in aqueous solutions

**DOI:** 10.3389/fbioe.2023.1253652

**Published:** 2023-10-10

**Authors:** Gabriele Sass, Marie-Christine Groleau, Eric Déziel, David A. Stevens

**Affiliations:** ^1^ California Institute for Medical Research, San Jose, CA, United States; ^2^ Institut National de la Recherche Scientific-Centre Armand-Frappier Santé Biotechnologie, Laval, QC, Canada; ^3^ Division of Infectious Diseases and Geographic Medicine, Department of Medicine, Stanford University School of Medicine, Stanford, CA, United States

**Keywords:** biosurfactant, quantification, assay, rhamnolipids, *Pseudomonas*, *Burkholderia*

## Abstract

Biosurfactants are microbial products that have applications as cleaning agents, emulsifiers, and dispersants. Detection and quantification of biosurfactants can be done by various methods, including colorimetric tests, high performance liquid chromatography (HPLC) coupled to several types of detectors, and tests that take advantage of biosurfactants reducing surface tension of aqueous liquids, allowing for spreading and droplet formation of oils. We present a new and simple method for quantifying biosurfactants by their ability, on paper, to reduce surface tension of aqueous solutions, causing droplet dispersion on an oiled surface in correlation with biosurfactant content. We validated this method with rhamnolipids, surfactin, sophorolipids, and ananatoside B; all are anionic microbial surfactants. Linear ranges for quantification in aqueous solutions for all tested biosurfactants were between 10 and 500 µM. Our method showed time-dependent biosurfactant accumulation in cultures of *Pseudomonas aeruginosa* strains PA14 and PAO1, and *Burkholderia thailandensis* E264. Mutants in genes responsible for surfactant production showed negligible activity on oiled paper. In summary, our simple assay provides the opportunity to quantify biosurfactant contents of aqueous solutions, for a diversity of surfactants, by means readily available in any laboratory.

## Introduction

Surfactants are surface-active compounds of a low molecular weight that can reduce surface tension of liquids, and can be used as emulsifiers, cleaning agents, and dispersants (Eras-Muñoz et al., 2022). In contrast to synthetic surfactants, biosurfactants are regarded to be more environmentally friendly, as they are biodegradable, and can be derived from renewable sources (Eras-Muñoz et al., 2022).

A variety of microorganisms produce surface-active agents to solubilize and use hydrophobic substances, such as oils or hydrocarbons, as carbon sources (Saeed et al., 2022; Sah et al., 2022), to promote group surface behaviour (Nielsen et al., 2002; Tremblay et al., 2007; Kearns, 2010; Nickzad et al., 2015; Morin et al., 2022) and act as antagonistic (Zulianello et al., 2006; Sidrim et al., 2020; Sass et al., 2021; Sass et al., 2022) and virulence factors (Zulianello et al., 2006; Köhler et al., 2010).

Classical methods of quantifying biosurfactants encompass oil displacement test, emulsion stability testing, droplet size analysis, critical micelle concentration (CMC) determination, high performance liquid chromatography (HPLC), and colorimetric tests, such as the methylene blue test, or the Congo Red test (Heyd et al., 2008; Jiang et al., 2020). Critical reviews of biosurfactant research and methodology have been published (Heyd et al., 2008; Twigg et al., 2021). A compressed overview of methods to evaluate the biosurfactant content in liquids is shown in Supplementary Table S1.

We took advantage of surface tension reduction by biosurfactants and applied aqueous solutions to a stationary layer of oil, here oiled paper on a glass surface, to create a very simple method for measurement. Quantification of biosurfactants by this method can be done very easily by determination of areas of dried droplets, and use of standard curves. Concentration of biosurfactants in aqueous solutions is equivalent to their droplet size over a wide range of concentrations.

## Materials and methods

### Materials

Natural *P. aeruginosa* rhamnolipids mixture (Rhl), *Bacillus* sp. surfactin (SF), methanol (MeOH), dimethylsulfoxide (DMSO), and RPMI 1640 medium were purchased from Sigma-Aldrich (St. Louis, MO). A *S. bombicola* sophorolipid mixture (SPL) was provided by Dispersa (Laval, Québec, Canada). Ananatoside B (AnaB) was synthetized by the group of Charles Gauthier (INRS, Laval, Québec, Canada) (Cloutier et al., 2021). The main rhamnolipid congener RhaRhaC14C14 of *B. thailandensis* was purified from cultures as described previously for *B. glumae* (Costa et al., 2011).

### Preparation of biosurfactant solutions

Rhl and SPL were dissolved and diluted in sterile water. SF and RhaRhaC14C14 were dissolved in sterile water, containing MeOH at 25% or 20%, respectively. Stock solutions were further diluted in water, so that all test concentrations contained 10% MeOH. AnaB was dissolved in DMSO. Test concentrations contained 10% DMSO. MeOH up to 12.5% and DMSO up to 25% to water did not affect area sizes of biosurfactant droplets in oiled paper assays. For standard curves, test concentrations of 1–500 µM were used except for surfactin for which concentrations of 1.2–300 µM were used.

### Biosurfactant quantification on oiled paper

Standard printer paper (20-pound type) was placed on a glass plate, which had been cleaned with 70% alcohol to achieve fat-free conditions. The paper sheet was evenly covered with at least 5 ml of fluid mineral oil (sterile mineral baby oil; CVS, Woonsocket, RI). Light mineral oil was best suitable for the assay. We did not compare brands. The amount of oil used will depend on the size and quality of the paper sheet used. Excess oil and air bubbles underneath the oiled paper were brushed off, using lint-free tissue paper (Kimtech, Kimberley-Clark Worldwide, Inc., Roswell, GA). Equal distribution of oil and removing excess oil until the paper surface no longer is shiny are measures to ensure comparability of measurements on different sheets, as is the use of background medium controls on each sheet. Fifty µl droplets of test samples were placed on oiled paper and allowed to spread and dry at room temperature. As controls, 50 µl droplets of sterile diluent, corresponding to the analyte, were placed on the same paper sheet. Diameters of dried droplets were measured vertically and horizontally, as drop shapes tended to be slightly irregular at higher biosurfactant concentrations. Diameters were used to calculate droplet areas, using the formula: a (area) = *r*(radius)2π. Mean areas of sterile diluent were subtracted from areas produced by biosurfactant suspensions. Areas are displayed in mm^2^.

### Facilitation of biosurfactant determination in water on oiled paper by addition of dye

**FIGURE 1:**
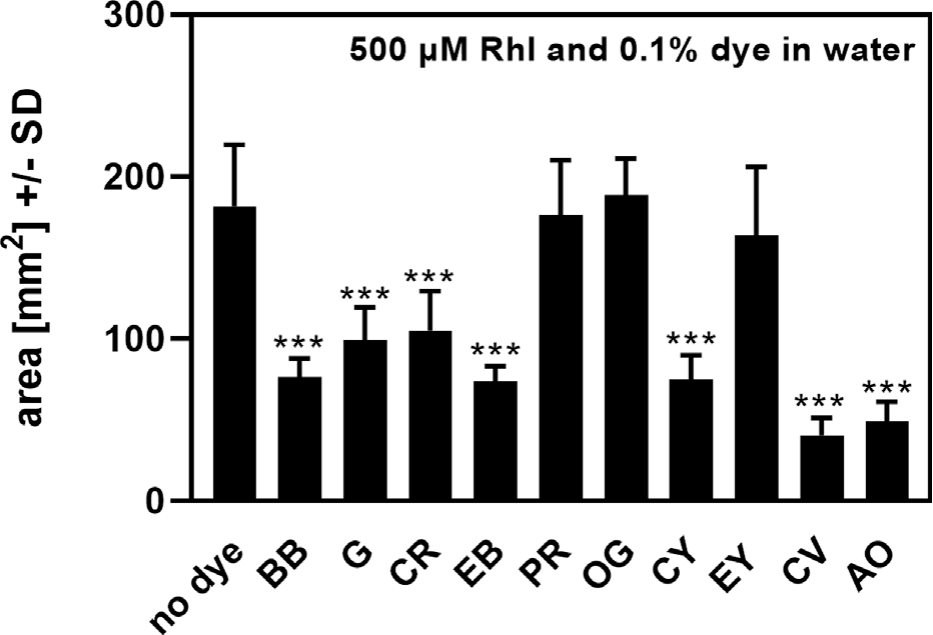
Effects of dyes on rhamnolipids measurement on oiled paper. Areas, produced by rhamnolipids (500 µM) in water, were determined by oiled paper assay with (0.1%) or without addition of dyes. Comparison: areas without added dye vs. areas containing dyes. Statistical analysis by *t*-test: Three asterisks = *p* ≤ 0.001. *n* = 8 technical replicates. Abbreviations: Rhl, rhamnolipids; BB, Brilliant Blue; G, Giemsa; CR, Congo Red; EB, Evans Blue; PR, Phenol Red; OG, Orange G; CY, Clayton Yellow; EY, Eosin Y; CV, Crystal Violet; AO, Acridine Orange.

When biosurfactants were examined in water, areas of droplets after drying were barely visible. To circumvent this problem, we tested a variety of dyes to add to our test samples (Table 1). Dyes shown in Table 1 were added to water, containing 0.5 mM Rhl, at concentrations of 0.1%, and a biosurfactant assay was performed on oiled paper. After determination of dried droplet areas, we found that three of the dyes, Phenol Red (PR), Orange G (OG), and Eosin Yellow (EY) did not interfere with Rhl measurement in water, compared to water alone (Figure 1). Of these dyes we used PR, subsequently at 0.01%, to facilitate measurement of anionic biosurfactant content in water, or water, containing MeOH or DMSO.

**TABLE 1:** Dyes tested as possible additives to water for this study.

Dye name	Abbreviation	Color	Molecular weight	Charge
Brilliant Blue	BB	Blue	792.9	Anionic
Giemsa	G	Violet	291.8	Neutral
Congo Red	CR	Red	696.7	Anionic
Evans Blue	EB	Blue	960.8	Anionic
Phenol Red	PR	Red	354.4	Anionic
Orange G	OG	Orange	452.4	Anionic
Clayton Yellow	CY	Yellow	695.7	Anionic
Eosin Y	EY	Red	691.9	Anionic
Crystal Violet	CV	Violet	408.0	Cationic
Acridine Orange	AO	Orange	265.35	Cationic

### Microorganisms

Bacterial strains used in this study are shown in Table 2. The use of all microbes in our laboratory is approved by the CIMR Biological Use Committee (approval no. 001-03Yr.17).

**TABLE 2:** Bacterial strains and mutants used in this study.

Bacteria	Strain	Description	Ref
*Pseudomonas aeruginosa*	PA14	UCBPP-PA14; wild-type strain; common laboratory strain	O’Toole and Kolter (1998), Lee et al. (2006), Fischer et al. (2016)
*P. aeruginosa*	PA14*rhlA*	*rhlA*::MrT7, GmR	O’Toole and Kolter (1998), Lee et al. (2006), Fischer et al. (2016)
*P. aeruginosa*	PAO1	Wildtype, common laboratory strain	O’Toole and Kolter (1998), Lee et al. (2006), Fischer et al. (2016)
*Burkholderia thailandensis*	E264	Wildtype strain	Brett et al. (1998), Dubeau et al. (2009)
*B. thailandensis*	E264*rhlA*-	*rhlA1*::pKnock-Tc, *rhlA2*::*dhfrII*, TcR, tpR	Brett et al. (1998), Dubeau et al. (2009)

Gm, gentamicin; Tc, tetracycline; Tp, trimethoprim.

### Culture production

Bacteria (5 × 10^7^ cells/ml) or yeast (5 × 10^5^ cells/ml) were incubated at 35°C and 100 rpm for 24–96 h. *Pseudomonas* and *Burkholderia* strains were cultured in RPMI 1640 medium. RPMI was selected because it is completely defined, and, with additives, can be used in future experiments that require co-culture with mammalian cells, since RPMI can sustain mammalian cell growth.

### Preparation of bacterial filtrates

*P. aeruginosa* filtrates were prepared as detailed previously (Ferreira et al., 2015). Cultures were centrifuged at 200 g for 30 min at room temperature to produce supernatants, which were filtered for sterility (0.22 μm) using mixed cellulose ester (MCE), or cellulose acetate (CA) filters (Celltreat Scientific Products, Pepperell, MA).

### Statistical analysis

Results were analyzed using one way ANOVA or Student’s t-test. All data in this study are expressed as a mean ± SD. Each assay was performed with at least 3 biological and 8 technical replicates. Representative experiments are shown.

## Results

### Correlation between area size on oiled paper and biosurfactant concentration in aqueous liquid

**FIGURE 2:**
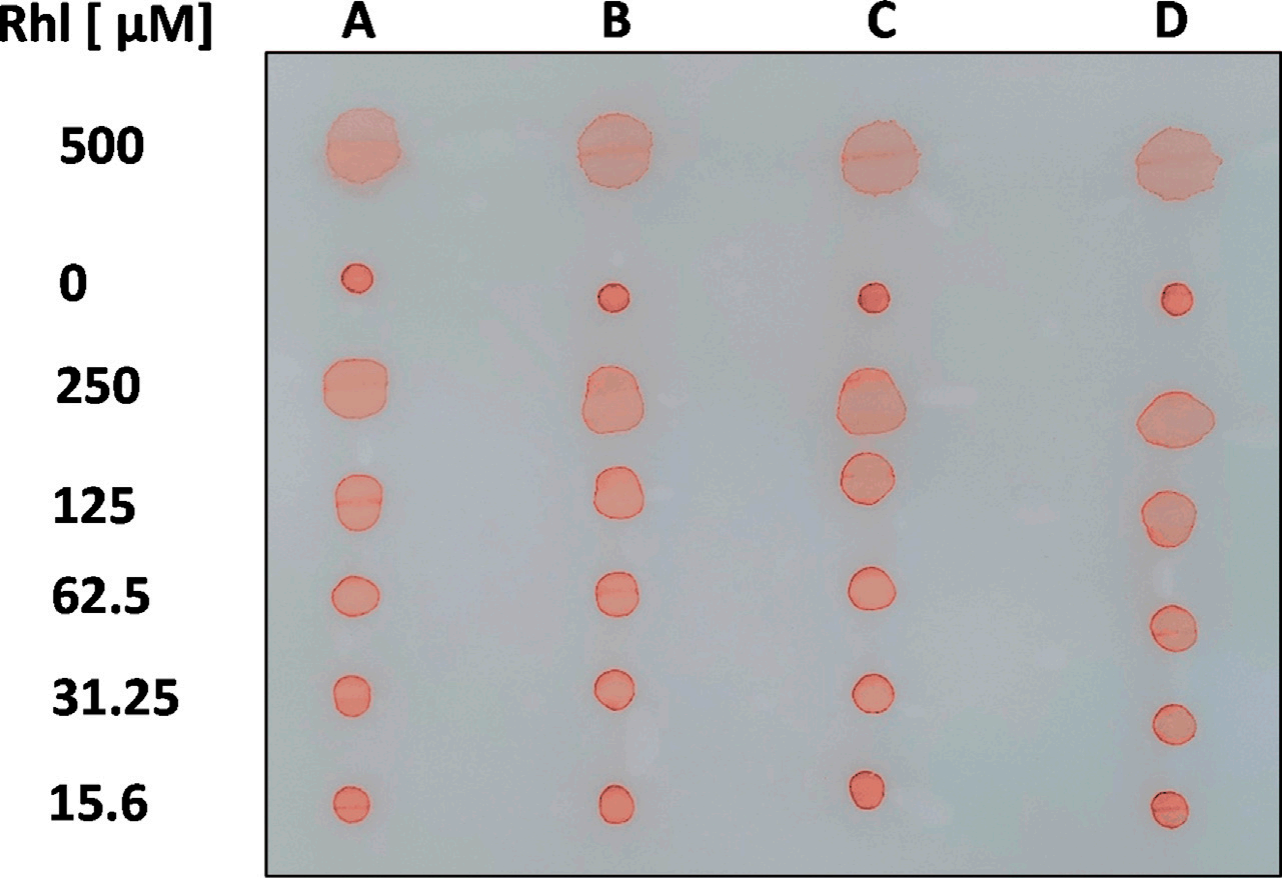
Visualization of the assay. Four 1:2 dilution rows **(A–D)** for rhamnolipids (500–15.6 µM; Rhl) in aqueous solution, containing PR (0.01%), were spotted on oiled paper, along with a background control (diluent with 0.01% PR). After droplets dried, a picture was taken.

To prepare standard curves for Rhl, RhaRhaC14C14, SF, SPL, and AnaB, aqueous dilutions of these anionic biosurfactants, containing 0.01% PR, were spotted on oiled paper, and allowed to dry at room temperature. Some of our biosurfactant stocks were prepared in MeOH or DMSO (see Materials and Methods). We found that in water, including MeOH up to 12.5% or DMSO up to 25% did not affect area sizes of biosurfactant droplets in oiled paper assays. Figure 2 illustrates our assay, showing areas of four aqueous dilution rows for Rhl (A-D), containing 0.01% PR, in comparison to the diluent (here: water) background controls, also containing 0.01% PR (0 µM Rhl). Means of background control areas were subtracted from areas generated by biosurfactant-containing solutions. In all assays, droplet diameters critically were measured in horizontally in two perpendicular axes, as concentrations of biosurfactants ≥125 µM tended to produce irregular droplet shapes (Figure 2).

**FIGURE 3:**
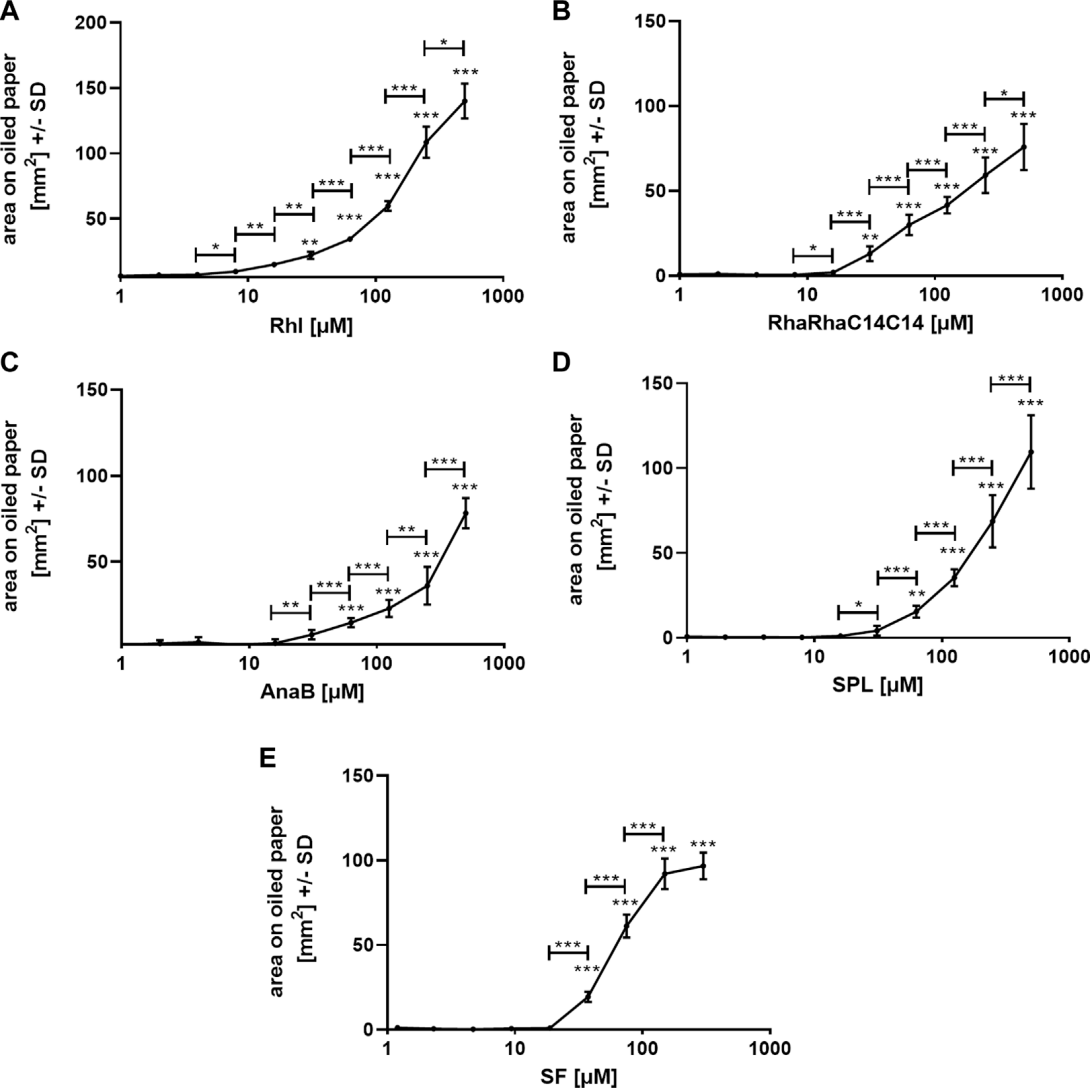
Standard curves on oiled paper for biosurfactants in water. Standard curves for rhamnolipids [**(A)**; 1–500 μM; Rhl], RhaRhaC14C14 [**(B)**; 1–500 µM], ananatoside B [**(C)**; 1–500 μM; AnaB], sophorolipids [**(D)**; 1–500 μM; SPL], and surfactin [**(E)**; 1.2–300 μM; SF] in aqueous solution, containing PR (0.01%) were established by spotting 1:2 dilutions, or background control (sterile water with 0.01% PR) on oiled paper. After droplets dried, diameters were determined, areas calculated, and background areas were subtracted from areas produced by biosurfactant dilutions. Resulting areas were plotted against biosurfactant concentrations. Comparisons without brackets by one way ANOVA: 1 µM vs. all other concentrations. Other comparisons by *t*-test, as indicated by the ends of the brackets. Statistical analysis: One, two, or three asterisks = *p* ≤ 0.05, *p* ≤ 0.01, or *p* ≤ 0.001, respectively. *n* = 10 technical replicates.

We used one oiled paper sheet per biosurfactant and repetition experiment, with each sheet containing its own background control. All experiments were repeated at least twice, and Figure 3 shows representative results for each biosurfactant. The lowest Rhl (Figure 3A), or RhaRhaC14C14 concentration (Figure 3B) with significantly larger area on oiled paper than 1 µM was 31 µM (*p* ≤ 0.01). For AnaB (Figure 3C) and SPL (Figure 3D) this concentration was at 63 μM, and for SF this concentration was at 37.5 µM (Figure 3E). Comparison of neighboring concentrations revealed significant differences starting between 4 and 16 µM and lasting up to at least 250 µM (Figure 3). SPL (Figure 3D) and AnaB (Figure 3E) still showed no flattening of the curve even between 250 and 500 µM.

### Biosurfactant production in cultures of *P. aeruginosa* and *B. thailandensis* is detected using our method

**FIGURE 4:**
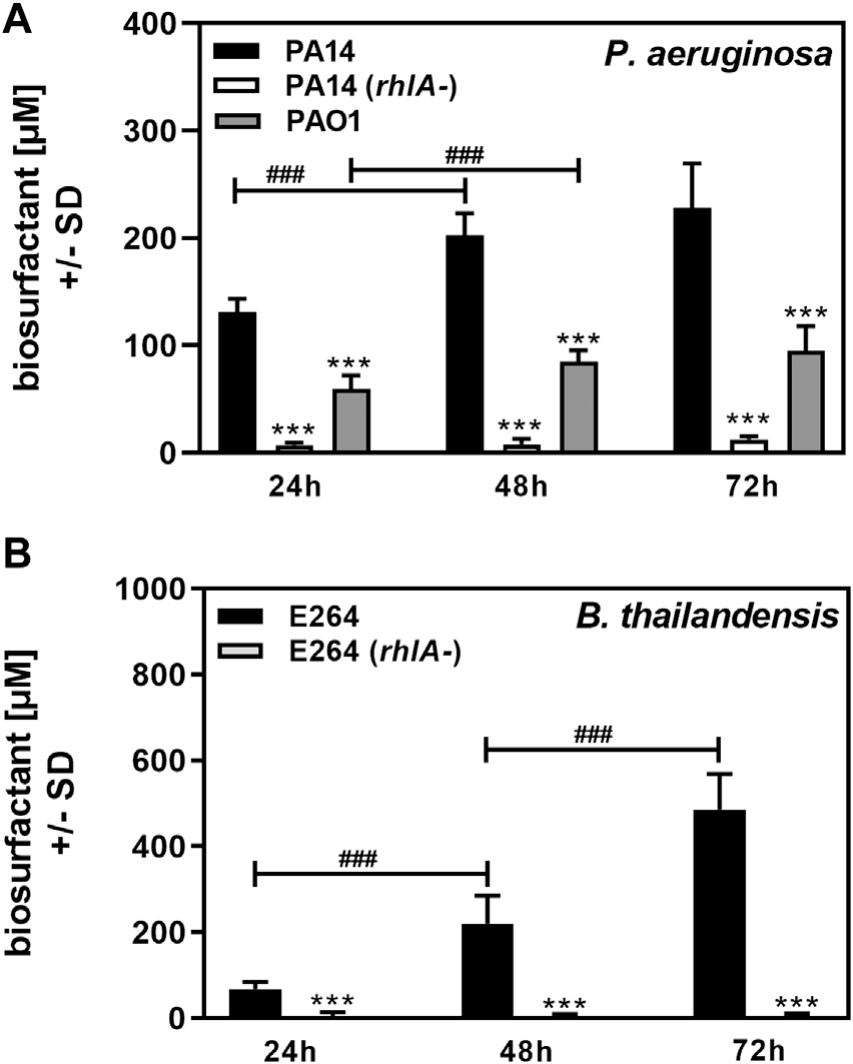
Determination of biosurfactant amounts in planktonic cultures. Cultures of *P. aeruginosa* wildtype (PA14, PAO1), or a PA14 *rhlA* mutant **(A)**, or *B. thailandensis* wildtype (E264), or an E264 *rhlA* mutant **(B)** were prepared in RPMI medium, starting from 5 × 10^7^ bacteria/ml. Cultures were incubated at 37°C. At 24, 48, and 72 h, cultures were analyzed for biosurfactant production on oiled paper. Diameters of dispersed 50 µl droplets from culture suspensions were determined and used for the calculation of areas. Areas produced by sterile medium were subtracted from areas produced by culture suspensions. Areas were used to calculate biosurfactant content using the respective standard curves in Figure 3. Comparisons without brackets: Wildtype vs. all other strains at the same timepoint. Other comparisons as indicated by the ends of the brackets. Statistical analysis by *t*-test: One, two, or three asterisks or pound signs = *p* ≤ 0.05, *p* ≤ 0.01, or *p* ≤ 0.001, respectively. Pound signs indicate significant increases, asterisks indicate significant decreases. **(A)**: *n* = 12 technical replicates; **(B)**: *n* = 8 technical replicates.

*P. aeruginosa* is the best-known producer of Rhl (Soberón-Chávez et al., 2005). As a proof of principle for our method we used bacterial cultures of *P. aeruginosa* wildtype strain PA14 (O’Toole and Kolter, 1998; Lee et al., 2006; Fischer et al., 2016), its *rhlA*-mutant, unable to produce Rhl (Ochsner et al., 1994), and a second *P. aeruginosa* wildtype strain, PAO1 (Stover et al., 2000). We also studied *B. thailandensis*, the producer of the higher molecular weight rhamnolipid RhaRhaC14C14 (Brett et al., 1998; Dubeau et al., 2009), as well as its *rhlA*-mutant (Brett et al., 1998; Dubeau et al., 2009). Planktonic cultures were grown from an equal number of cells in RPMI medium over a period of 72 h. Biosurfactant content in planktonic cultures was determined at 24, 48, and 72 h. We found that wildtype PA14 and PAO1 culture biosurfactant content significantly increased up to 48 h (Figure 4A). PAO1 cultures contained significantly less biosurfactants than PA14 cultures (Figure 4A). The assay detected small amounts of amphiphilic molecules in cultures of the PA14 *rhlA*-mutant (Figure 4A), confirming that the vast majority of surface-active molecules present in cultures of PA14 are Rhl. Using the standard curve shown in Figure 3A we determined that PA14 cultures in RPMI contained a mean of 131 µM Rhl at 24 h, 202 μM at 48 h, and 228 μM at 72 h. PAO1 cultures contained a mean of 60 µM Rhl at 24 h, 85 μM at 48 h, and 95 μM at 72 h.

*B. thailandensis* E264 culture biosurfactant content increased up to at least 72 h, whereas its rhlA-mutant produced only very small amounts of amphiphilic molecules, indicating that the major biosurfactants produced by *B. thailandensis* were Rhl (Figure 4B). Using the standard curve in Figure 3B we determined that E264 cultures in RPMI contained a mean of 68 µM Rhl at 24 h, 219 μM at 48 h, and 486 μM at 72 h.

### Filtration impacts biosurfactant content

**FIGURE 5:**
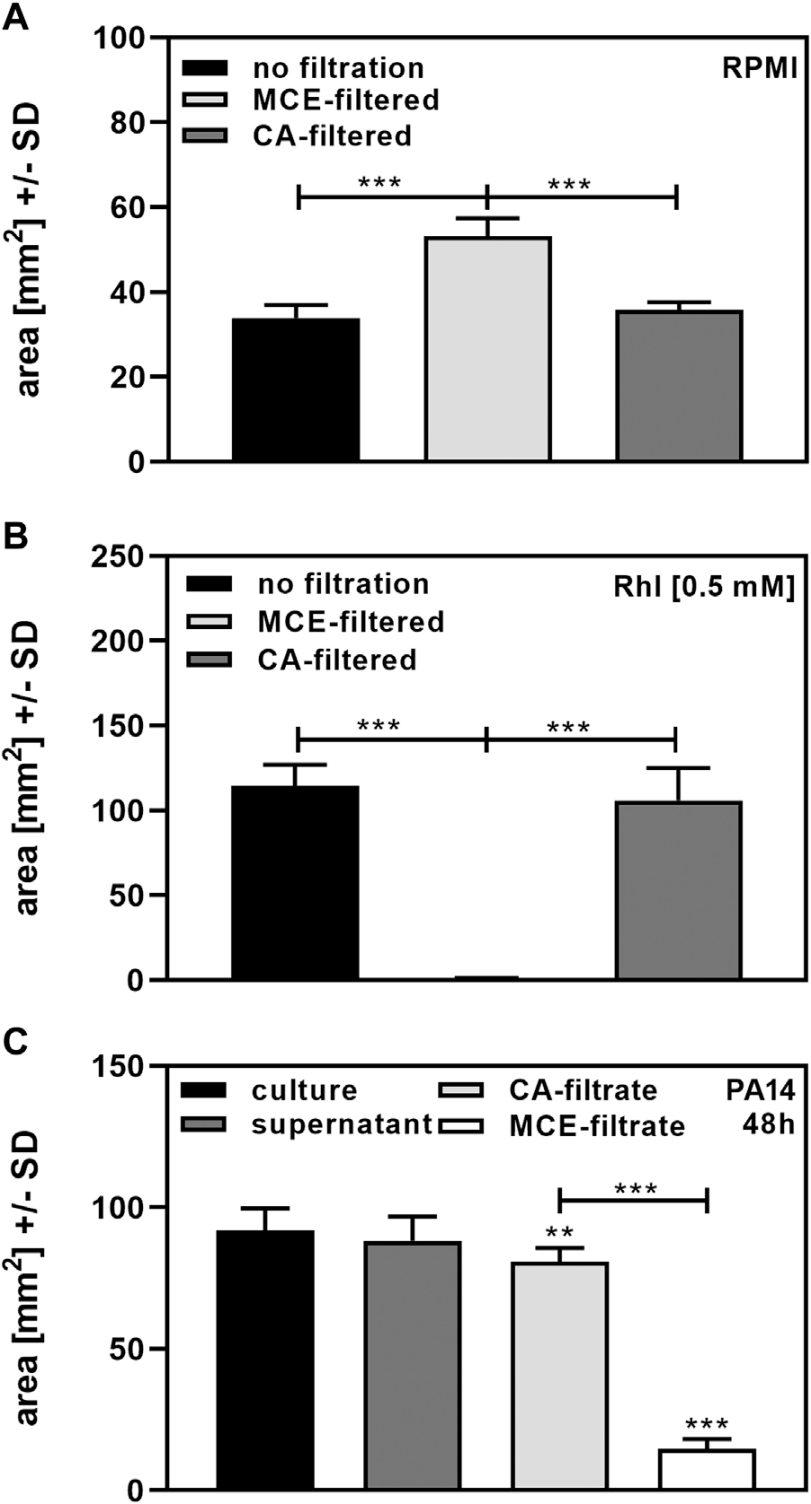
Determination of biosurfactants after filtration. **(A)**: Portions of sterile RPMI medium were filtered through 0.2 µm MCE or CA filters. Diameters of dispersed 50 µl droplets were determined and used for the calculation of areas. Comparisons as indicated by the ends of the brackets. **(B)**: 0.5 mM Rhl were added to sterile RPMI medium, and portions filtered through 0.2 µm MCE or CA filters. Diameters of dispersed 50 µl droplets from unfiltered RPMI or filtrates were determined and used for the calculation of areas. Comparisons as indicated by the ends of the brackets. **(C)**: Cultures of *P. aeruginosa* wildtype (PA14), were prepared in RPMI medium, starting from 5 × 10^7^ bacteria/ml. Cultures were incubated at 37°C for 48 h. Part of the culture was centrifuged to obtain supernatants, and two five ml portions of the supernatant were filtered through 0.2 µm MCE or CA filters. Diameters of dispersed 50 µl droplets from culture suspension, supernatant or filtrates were determined and used for the calculation of areas. Areas produced by sterile medium (unfiltered for culture or supernatant, or filtered through CA or MCE, respectively) were subtracted from areas produced by culture suspensions. Comparisons without brackets: Culture vs. all other bars. Other comparisons as indicated by the ends of the brackets. Statistical analysis by *t*-test: Two or three asterisks = *p* ≤ 0.01 or *p* ≤ 0.001, respectively. *n* = 8 technical replicates.

Finally, we investigated effects of centrifugation and filtration on biosurfactant measurement in our assay. Areas for RPMI after MCE filtration were significantly larger than areas following CA filtration, or when RPMI was not filtered through MCE or CA filters, suggesting that MCE filters released a surfactant into the filtrate (Figure 5A). When 0.5 mM Rhl was added to RPMI, we found that MCE-filtration removed almost all biosurfactant, whereas CA-filtration did not (Figure 5B). While cultures and their centrifuged supernatants after 48 h of incubation did not differ significantly in biosurfactant content, filtration of 5 ml supernatant through MCE removed 80% of biosurfactant content from cultures of PA14 (Figure 5C). Filtration of 5 ml supernatant through CA also had a significant, but much less pronounced effect on biosurfactant content (Figure 5C). Filter effects were even more pronounced when lower volumes were filtered, or supernatants tested that contained less biosurfactants, e.g., harvested after 24 h of culture. We therefore recommend avoiding filtration before using our biosurfactant assay when low concentrations of biosurfactants are present in cultures. If filtration is needed, filtration through CA filters is preferable to MCE filtration.

## Discussion

Many methods are available for measurement of amphiphilic glycolipids such as Rhl. These include, roughly from simplest to more sophisticated and precise: assay of hemolysis, the drop collapsing test, measurement of surface tension of a spreading drop on agar, anthrone or orcinol colorimetry, use of fluorescein-encapsulating vesicles, thin layer chromatography, high performance liquid chromatography (HPLC), and HPLC coupled with mass spectroscopy (LC/MS) (Abdel-Mawgoud et al., 2014; Kubicki et al., 2020; Twigg et al., 2021). In a previous study we compared the LC/MS method, after extraction from agar to an assay measuring rhamnolipid production by bacterial colonies on agar by oil displacement (Abdel-Mawgoud et al., 2014; Kubicki et al., 2020; Twigg et al., 2021). We found similar results between LC/MS and oil displacement assay on agar (Sass et al., 2021), indicating that oil displacement is a simple alternative to LC/MS for studying events on agar that quantifies, but not specifies, molecules. In contrast to LC/MS, measuring oil displacement on agar is a non-specific method only allowing semi-quantitative comparison of production of surface-active molecules between bacterial strains. Using the oil displacement method, we found that a double siderophore mutant produced more Rhl than its PA14 wildtype (Abdel-Mawgoud et al., 2014; Kubicki et al., 2020; Twigg et al., 2021).

Here we aimed at quantification of various biosurfactants in aqueous solutions, by use of a very simple method, potentially broadly available, in combination with a standard curve. We took advantage of the fact that surface tension of a water droplet declines with its surfactant content, and that this allows further spreading on a hydrophobic surface. The hydrophobic surface we used was a sheet of ordinary printer paper, on a glass surface, with the paper covered in a thin layer of light mineral oil. We found that on oiled paper, 50 µl droplets of aqueous solutions not containing surfactants spread widely enough to be easily measurable by a caliper. On non-oiled paper such droplets simply are absorbed over time, not leaving a measurable perimeter. When solutions contained biosurfactants we were able to produce a standard curve, suitable for measurement between about 10 and at least 250 µM. Higher concentrations of biosurfactants need to be diluted for accurate measurement. Uniformity of results for five biosurfactants by measurement on oiled paper also suggests that our method is universally applicable to biosurfactants, no matter the source. Both glycolipids and a lipopeptide were measurable.

When measuring biosurfactants in aqueous solutions, we found that adding color greatly facilitated the determination of dried drop diameters. All biosurfactants tested here were anionic, so we expected any anionic dye to be suitable. Anionic dyes are not expected to interact with anionic biosurfactants, but this proved to be not so simple. Of all anionic dyes tested here, only Phenol Red (PR), Orange G (OG) and Eosin Yellow (EY) were found to not interfere with biosurfactant measurements. We added PR to all further studies in water, as it already is part of RPMI medium, which we subsequently used for preparing bacterial cultures. PR concentrations in water of 0.01% were sufficient to produce easily measurable spots on oiled paper. RPMI, which contains 0.005% PR was used as supplied, without adding dye.

In all studies shown we used ordinary printer paper of 20-pound size. We compared that to higher quality paper and found that the resolution of our standard curves for high concentrations improved with paper quality, whereas the resolution for low concentrations declined. In principle, it depends on the biosurfactant content expected in cultures which paper quality is best suited for the individual assay, but it is essential that the same quality of paper is used for comparison of standard curve and test samples, and that a background control always is part of the assay.

Measurements of biosurfactant levels were performed from planktonic bacterial cultures using our new assay. *P. aeruginosa* PA14 cultures increased in biosurfactant content over 72 h, to a mean content of over 300 µM. These are biologically relevant concentrations, as the IC50 for Rhl on forming *A. fumigatus* 10AF biofilm is about 160 µM (Sass et al., 2021). PAO1, another wildtype laboratory strain of *P. aeruginosa*, produced less Rhl than PA14 under our conditions.

The *rhlA*-mutants of *P. aeruginosa* and *B. thailandensis* are unable to produce Rhl, but still produced small areas in our assay. As we used bacterial cultures, containing cells, it is likely that amphiphilic molecules, fatty acids or various membrane lipids, such as LPS, are detected by our method, creating a minimal background.

Overall, the choice of medium affected the output of biosurfactants. The media used here were chosen, because they are used in our laboratory, and others, in various projects, and are fully defined. Other media might allow production of different yields of biosurfactants. For example, we have studied trypticase soy broth medium as a growth medium and found biosurfactant yield quantitatively different from the media studied here (data not shown). Culture conditions can also affect biosurfactant yield: we have studied cultures in hypoxic conditions, and found, provided normalization to bacterial growth is done, there was equivalence in biosurfactant yield (data not shown). It is important to be cognizant of the course of biosurfactant production, when determining the time for reading results.

We observed that filtration of RPMI through CA filters did not remove added Rhl, or washed unrelated surfactants from filters into filtrates, as observed for MCE filters. MCE filtration almost completely removed biosurfactants from cultures, whereas CA filtration removed 10%–50%, depending on the volume filtered, and the concentration of biosurfactants in supernatants. We recommend determining biosurfactants in supernatants without prior filtration. Cells and debris should be removed by centrifugation, and supernatants studied. However, in some of the assays we report here, use of un-sedimented culture did not give different results than the use of supernatants. Adding certain dyes, e.g., PR, to test liquids facilitates biosurfactant measurement without affecting quantification, and is recommended.

In summary, although we only tested a limited number of biosurfactants (but including the best known), we predict that our simple method of biosurfactant quantification can be applied to a wide variety of either purified biosurfactants, or biosurfactants from cultures, or culture supernatants, and is qualitative or semiquantitative. Supplementary Table S2 compares the established but sophisticated method of HPLC to our simple method. The main advantage of our method is that it does not require expensive equipment and highly skilled personnel, does not require extraction, but still can compare biosurfactant production between related strains, or the same strain in different media. The method also will be very useful in screening for new biosurfactant producers, and with high throughput. In cultures producing only one biosurfactant, and in combination with a standard curve, our method can provide quantitative data.

## Data Availability

The raw data supporting the conclusion of this article will be made available by the authors, without undue reservation.

## Abbreviations

Rhl, rhamnolipids; SPL, sophorolipids; SF, surfactin; AnaB, ananatosideB; BB, Brilliant Blue; G, Giemsa; CR, Congo Red; EB, Evans Blue; PR, Phenol Red; OG, Orange G; CY, Clayton Yellow; EY, Eosin Y; CV, Crystal Violet; AO, Acridine Orange; MCE, mixed cellulose ester; CA, cellulose acetate.
