# Supplementary material for: Simple method for quantification of anionic biosurfactants in aqueous solutions
Source: Front Bioeng Biotechnol. 2023 Oct 10;11:1253652. doi: 10.3389/fbioe.2023.1253652 (PMC10598384; doi:10.3389/fbioe.2023.1253652)
Supplement: Supplementary file 1 [file Table1.pdf]

Supplemental Tables

Supplementary Table 1: Comparison of methods used to measure biosurfactant content in liquids

Method	Principle	Accuracy and resolution	Information	Complexity
Mass spectrometry	Identifies and quantifies biosurfactant molecules based on their mass-to-charge ratio	Very high	Identification and quantitative analysis	Requires advanced equipment and expertise, and prior knowledge of the nature (mass or m/z) of the biosurfactant
High-Performance Liquid Chromatography (HPLC)	Separates biosurfactant compounds and determines their concentration in liquid samples, if a detector is coupled.	High	Quantitative	Requires advanced equipment and expertise
Thin Layer Chromatography (TLC)	Separates and detects different biosurfactants based on their chemical properties	High	Identification and semi-quantitative analysis	Used to assess the presence of already known congeners. Requires specialized equipment and expertise.
Surface Tension measurement (STM)	Reduction of surface tension of liquids by biosurfactants	High	Quantitative, but indirect, requiring knowledge of the critical micelle concentration	Requires specialized equipment
Emulsification Activity (EA)	Biosurfactants have the ability to emulsify hydrocarbons or other immiscible liquids	Medium	Indirect, but can be quantitative	Requires specific emulsion systems
Oil spreading	Oil-displacement by biosurfactant-	Medium	Qualitative, can be semiquantitative	Requires some expertise

	containing liquids			

Supplementary Table 2: Comparison of HPLC vs biosurfactant measurement on oiled paper

**Method**	**High-Performance Liquid Chromatography (HPLC)**	**Biosurfactant measurement on oiled paper**
**Principle**	Quantifies biosurfactant compounds and determines their concentration in liquid samples.	Defined drops of biosurfactant-containing liquid spread on oiled paper proportionally to their biosurfactant content
**Specificity and resolution**	High	Medium
**Information**	Qualitative and quantitative, can show congeners if coupled with mass spectroscopy	Qualitative, quantitative with standard curve.
**Limitations**	Requires advanced equipment and expertise	Simple
**Applications**	Chromatography technique for separation, identification, and quantification of biosurfactants in a mixture when combined with an appropriate detector	Screening method for relative amounts of biosurfactants in different batches of production; can be used for quantitative analysis if the composition of the biosurfactant produced by the microbial culture is known, and the respective molecule is available to prepare a standard curve.
